# A rare large homozygous deletion of the *HFE* gene in a patient with hemochromatosis: a case report

**DOI:** 10.1016/j.htct.2026.106505

**Published:** 2026-08-06

**Authors:** Roya Gasimli, Serife Solmaz, Asli Subasioglu

**Affiliations:** a Ege University Faculty of Medicine, Department of Medical Biology, Izmir, Turkey; b İzmir Kâtip Çelebi University, Department of Hematology, Atatürk Training and Research Hospital, İzmir, Turkey; c Izmir Katip Celebi University, Department of Medical Genetics, Ataturk Training and Research Hospital, Izmir, Turkey

**Keywords:** Hemochromatosis, HFE gene, Homozygous deletion, Next generation sequencing, Chromosomal microarray analysis

## Introduction

Hemochromatosis is a systemic condition marked by excessive iron deposition across multiple tissues and organs, resulting from uncontrolled intestinal iron absorption caused by altered levels of the peptide hormone hepcidin or, more rarely, resistance to hepcidin, which regulates iron homeostasis. Hemochromatosis is a genetically heterogeneous disorder, encompassing several distinct forms dictated by specific genetic variations. Recent classification frameworks define hemochromatosis as a heterogeneous disorder comprising *HFE*-related, non–*HFE*-related, digenic, and molecularly undefined forms. The *HFE*-related subtype most frequently results from pathogenic variants in the *HFE* gene, typically p.Cys282Tyr homozygosity or compound heterozygosity with other rare variants, including large *HFE* deletions. This form is characterized by variable penetrance, with host-specific and environmental factors contributing to the development of iron overload. In contrast, non–HFE-related hemochromatosis is caused by rare pathogenic variants in genes involved in hepcidin regulation, such as the *HJV, HAMP, TFR2*, and *SLC40A1* genes. Digenic hemochromatosis involves pathogenic variants affecting more than one gene implicated in iron metabolism, whereas molecularly undefined cases lack an identifiable genetic cause despite comprehensive sequencing of known hemochromatosis-associated genes [1].

The HFE gene, located on chromosome 6 (6p22.2) within the major histocompatibility complex region, encodes the HFE protein, which is structurally similar to major histocompatibility complex class I proteins. The HFE protein is responsible for regulating iron absorption by modulating the interaction between transferrin and the transferrin receptor [2]. *HFE-*related hemochromatosis is associated with various mutations in the *HFE* gene, including common single nucleotide polymorphisms (SNPs) such as p.His63Asp and p.Cys282Tyr, as well as rare copy number variations (CNVs) [3]. Although the p.Cys282Tyr mutation is the most common pathogenic variant, its frequency is markedly lower in certain populations, including Brazilian populations [4]. These mutations, when present in a homozygous state due to the autosomal recessive inheritance pattern, may result in structurally abnormal or non-functional protein products and can be associated with clinical indicators such as increased ferritin levels (typically >300 µg/L in males).

Excessive iron accumulation can lead to various complications, including cirrhosis, liver cancer, diabetes, cardiomyopathy, joint disorders, and hormonal imbalances due to reduced gonadotropin levels.

There are several commonly used genetic diagnostic methods, including Sanger sequencing for specific targeted regions; Next Generation Sequencing (NGS) for single or multigene panels to detect SNPs and small CNVs; and techniques such as multiplex ligation-dependent probe amplification (MLPA) or chromosomal microarray analysis (CMA) for identifying large deletions and duplications.

This patient was referred from the hematology department due to elevated serum ferritin levels (711 µg/L). NGS was performed using a custom HFE gene panel, and the resulting library was sequenced on the Illumina MiSeq platform. Consequently, zero coverage was observed across all triplicate samples. Consequently, we suspected the presence of a large deletion that remained undetected due to the inherent limitations of short-read NGS. Subsequently, CMA, the primary modality available for copy number assessment in our laboratory, detected a 6863-bp homozygous deletion within the HFE gene. This CNV is presumed to be pathogenic for HFE-related hemochromatosis and represents a novel or exceedingly rare variant in the literature.

## Case presentation

A 31-year-old male patient was referred from the hematology department due to elevated serum ferritin levels. His medical history is notable for a prior thyroid lobectomy performed for papillary thyroid carcinoma. Abdominal imaging revealed Grade 1 hepatosteatosis. The patient's parents are consanguineous (first cousins), a factor that elevates the probability of homozygous hereditary conditions.

In order to better characterize the patient’s condition, detailed hematological, biochemical, and molecular investigations were carried out. Particular attention was given to iron metabolism markers, as both ferritin and transferrin saturation levels were markedly elevated. The relevant findings are presented in Table 1.

**Table 1:** Patient’s blood tests results.

Test	Value	Reference Range	Interpretation
Hemoglobin	17.1 g/dL	12–16 g/dL	↑ (Polycythemia)
Hematocrit	47%	36–46%	Slightly ↑
WBC	7.16 × 10⁹/L	4–10 × 10⁹/L	Normal
Neutrophils	4.32 × 10⁹/L	2–7 × 10⁹/L	Normal
Platelets	247 × 10⁹/L	150–450 × 10⁹/L	Normal
AST	Normal	0–40 U/L	–
ALT	Normal	0–40 U/L	–
Bilirubin	Normal	0.1–1.2 mg/dL	–
Creatinine	Normal	0.6–1.2 mg/dL	–
Fasting glucose	Normal	70–100 mg/dL	–
Serum iron	196 µg/dL	70–180 µg/dL	↑
TIBC	284 µg/dL	150–450 µg/dL	Normal
Transferrin saturation	69%	20–50%	↑
Ferritin	717 µg/L	30–300 µg/L	↑
Serum EPO	5.9 mIU/mL	4.3–29 mIU/mL	Low-normal
JAK2 V617F	Negative	–	Excludes PV

WBC: White blood cells; AST: Aspartate aminotransferase; ALT: Alanine aminotransferase; TIBC: Total iron-binding capacity; EPO: Erythropoietin.

During the primary analysis and quality control assessment, no coverage was obtained for the *HFE* gene. To rule out technical errors, the sequencing was repeated in triplicate using the same DNA sample and once using newly extracted DNA from a second peripheral blood draw. However, all attempts consistently resulted in zero coverage for the *HFE* gene region. Due to the limitations of short-read NGS in detecting large deletions, we suspected a structural variant in this gene and thus proceeded with CMA.

**Figure 1:**
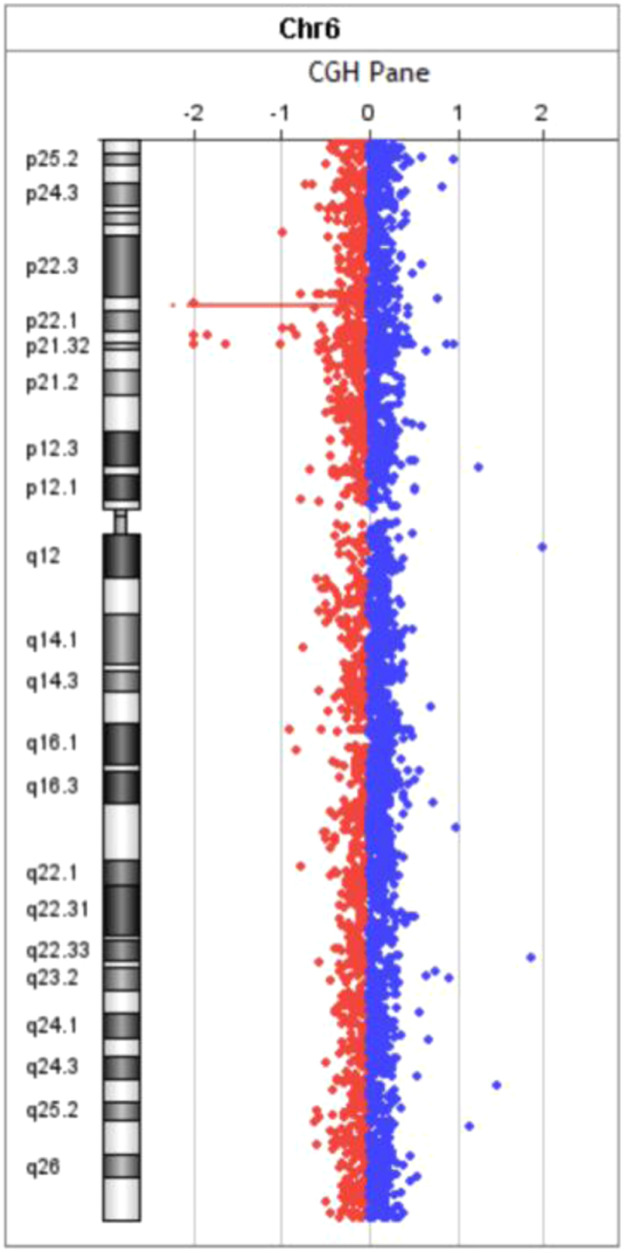
CMA profile of chromosome 6 indicating a copy number loss (red) at the HFE gene locus (6p22.2).

**Figure 2:**
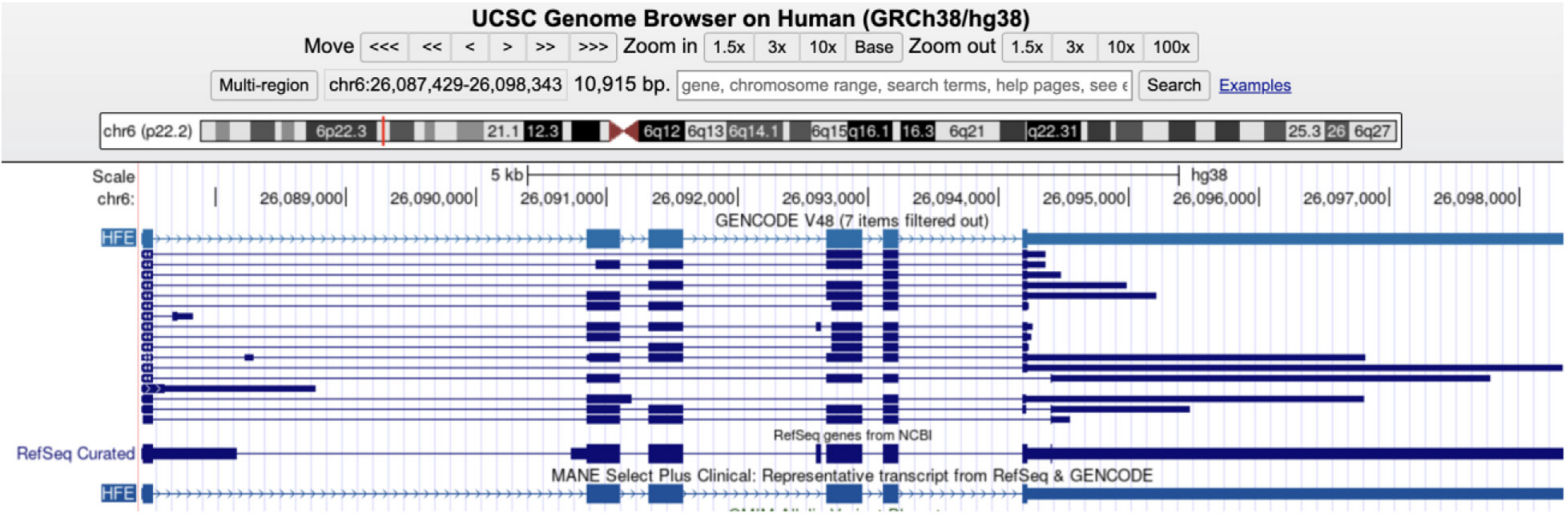
UCSC Genome Browser representation of the HFE gene locus on chromosome 6 (GRCh38).

Array-based comparative genomic hybridization analysis revealed a 6.863 kb deletion at chromosome 6p22.2 (chr6:26,088,186–26,095,048; GRCh38) (Figure 1), encompassing exons 2–5 and part of exon 6 of the *HFE* gene, while exon 1 remained intact (Figure 2). This structural alteration eliminates critical coding regions, including the positions of common pathogenic variants (p.His63Asp in exon 2 and p.Cys282Tyr in exon 4). Consequently, it is predicted to result in a complete loss of HFE function. This loss is characteristically associated with HFE-related hemochromatosis, and the variant has been classified as pathogenic.

The patient presented without a history of alcohol use or signs of metabolic syndrome. In cases of severe iron overload, thorough cardiac examinations are indicated to monitor for complications such as dyspnea. Additionally, systemic evaluations of bone, joint, and endocrine functions should be tailored to the patient's specific clinical presentation. However, this patient had no symptoms that would necessitate cardiac, joint, or endocrine studies.

Phlebotomy should be offered to patients with *HFE*-related hemochromatosis and elevated serum ferritin levels (>300 µg/L in men). The patient was followed up for phlebotomy treatment in the hematology clinic.

## Discussion

Hemochromatosis is a genetic disorder characterized by excessive iron accumulation, resulting from mutations in genes that regulate iron metabolism. Among the four major subtypes, *HFE*-related hemochromatosis is the most common and is primarily associated with pathogenic variants in the HFE gene. While SNPs, such as p.Cys282Tyr and p.His63Asp, account for the majority of HFE-related cases, large deletions or CNVs involving this gene have also been reported, albeit rarely [5,6].

NGS is a widely used high-throughput technology that enables the analysis of targeted gene panels, whole exomes (WES), whole genomes (WGS), and transcriptomes [7]. However, conventional short-read NGS platforms have notable limitations in detecting structural variants such as large deletions or duplications, especially in guanine-cytosine-rich or repetitive regions. In such cases, alternative approaches like array-based comparative genomic hybridization, MLPA, or long-read sequencing technologies may be necessary to uncover clinically relevant genomic alterations that are missed by standard NGS pipelines.

In the present case, the patient exhibited markedly elevated ferritin levels, which serve as a strong indicator of underlying hemochromatosis. Therefore, the patient was referred from the hematology department to the Genetic Disorders Evaluation Center for targeted genetic testing, which included HFE gene sequencing and JAK2 mutation analysis. Based on these results, genetic counseling was provided.

According to the literature, *HFE*-related hemochromatosis can be caused by several common and diverse SNPs, as well as rare mutations.

This patient was referred for *JAK2* V617F mutation analysis and *HFE* gene sequencing. Similarly, Radwan et al. [8] previously reported a patient with JAK2-positive polycythemia vera who concurrently carried the HFE p.Cys282Tyr mutation, resulting in a dual diagnosis of polycythemia vera and HFE-related hemochromatosis. In contrast, this patient tested negative for the JAK2 V617F mutation, but molecular analysis revealed a large deletion in the HFE gene.

*HFE* gene deletions have been reported as one cause of hemochromatosis. A 47-year-old female with complete *HFE* loss showed moderate iron overload, while a 44-year-old male with a ferritin level of 2080 µg/L had absent mutation regions due to a genomic deletion. These findings suggest that large homozygous *HFE* deletions are more frequent in Sardinian and other Mediterranean populations [5]. This Turkish patient, presenting with a serum ferritin level of 717 µg/L, similarly demonstrates that germline homozygous *HFE* deletions can underlie hemochromatosis outside of traditionally high-prevalence geographic regions.

In the literature, the homozygous *HFE* splice-site mutation (IVS5+1 G>A) is known to cause severe iron overload and clinical hemochromatosis [9]. Consistent with those observations, the large deletion in this patient encompasses critical splice sites, mirroring the profound pathogenic impact associated with splicing disruption at this locus.

Previous literature has shown that homozygous deletions of the *HFE* gene may result in a clinical phenotype resembling that of the common homozygous *HFE* p.Cys282Tyr mutation, which is the most well-established pathogenic variant in hemochromatosis [6], particularly in Northern European populations **[**10], while its prevalence is lower in other regions such as in Brazilian populations [4]. On the other hand, a study conducted in a Turkish population demonstrated that the *HFE* p.His63Asp point mutation is more prevalent than the p.Cys282Tyr variant [11]. The present case involves a large deletion encompassing a substantial portion of the *HFE* gene, including exons 2 and 4, where the classic p.His63Asp and p.Cys282Tyr variants reside. This provides further evidence that structural variants disrupting this critical region drive an identical clinical phenotype. These findings suggest population-specific genetic heterogeneity in *HFE*-related hemochromatosis, with a potential role for structural variants.

According to the American College of Medical Genetics and Genomics (ACMG)/Association for Molecular Pathology (AMP) guidelines, the large homozygous deletion identified in this patient can be classified as pathogenic. This interpretation is supported by the presence of a loss-of-function event in the *HFE* gene (PVS1), its absence from population databases (PM2_Supporting), and the patient's strong phenotypic correlation with hemochromatosis (PP4) [12]. In line with previous reports, large deletions of the *HFE* gene are rare, but when present, they have a clear pathogenic impact on iron metabolism. p.Cys282Tyr homozygotes with raised serum ferritin levels (>200 µg/L in women; >300 µg/L in men) should undergo phlebotomy, whereas those without biochemical expression require surveillance and may be encouraged to donate blood voluntarily. The approach to maintenance therapy is individualized, with frequency determined by patient characteristics and initial ferritin values [13]. Patients with mildly elevated iron stores may still benefit from treatment. In particular, venesection in those with serum ferritin between 300 and 500 µg/L has been associated with improved outcomes, such as reduced cardiovascular events and decreased incidence of cancer [14].

During the maintenance phase, encouraging patients to participate in voluntary blood donation represents a practical and effective approach. Patients are often very interested in dietary treatments, but a diet low in iron has been shown to have little effect in lowering serum ferritin [14].

Proton pump inhibitors (PPIs) can reduce dietary iron absorption by suppressing gastric acid production; however, this effect is insufficient as a standalone treatment for hemochromatosis and cannot replace phlebotomy [15]. Finally, iron chelation therapy is rarely utilized, typically being reserved as an adjunct to phlebotomy in severe cases presenting with life-threatening cardiac dysfunction [16].

## Conclusion

The *HFE* deletion in the present case encompassed a large portion of the gene, including coding regions, with this finding correlating with the patient’s elevated ferritin levels. This case is noteworthy, as large deletions represent an exceedingly rare class of pathogenic variants within the *HFE* gene. From both our team’s experience and reports from other groups worldwide, partial or complete deletions of the *HFE* gene may have significant implications for the diagnosis and management of hemochromatosis, and future studies could help to refine their clinical interpretation and therapeutic relevance.

## Patient consent

The patient provided written informed consent for the publication of this case report.

## Author contributions

RG performed NGS and CMA experiments, analyzed the data, and drafted the manuscript. SS oversaw all hematology aspects and contributed to manuscript draft and revisions. AS supervised the study and provided overall guidance. All authors approved the final manuscript.

## Conflicts of interest

The authors report that they have no conflicts of interest related to this work.

## Data Availability

The data that support the findings of this study are available from the corresponding author upon reasonable request.
